# EEG-Based Evidence for Belief Coding Therapy in Treating Mental Disorders: A Cross-Sectional Interventional Study

**DOI:** 10.7759/cureus.93713

**Published:** 2025-10-02

**Authors:** Abhijeet Satani, Param Barodia

**Affiliations:** 1 Department of Neuroscience, Satani Research Centre, Ahmedabad, IND

**Keywords:** anxiety and depression, clinical remission, disorders, eeg monitoring, emotional control, mental health, neuroplasticity, neuropsychological treatment, therapeutic intervention, treatment-resistant depression

## Abstract

Background: Mental health disorders such as anxiety, depression, panic attacks, and trauma-related conditions are rising globally, presenting a significant public health challenge. Conventional treatments such as cognitive behavioural therapy (CBT) and pharmacological interventions offer symptom relief but often fail to address the underlying subconscious beliefs that perpetuate long-term emotional suffering. Belief Coding®, a novel neuropsychological therapy, was developed to identify and reprogram such limiting subconscious beliefs.

Methods: This study was a single-arm, mixed-methods investigation. A total of 75 participants diagnosed with anxiety, depression, or panic disorders were treated using Belief Coding®. Clinical outcomes were assessed with validated measures (GAD-7 (Generalized Anxiety Disorder-7), PHQ-9 (Patient Health Questionnaire-9), PDSS (Panic Disorder Severity Scale)), while neurophysiological changes were monitored via electroencephalography (EEG). The intervention included a structured, single-session protocol (~78 minutes) incorporating memory reconsolidation and multisensory anchoring.

Results: Significant symptom reductions were observed: GAD-7 anxiety scores decreased by 54.9%, PHQ-9 depression scores decreased by 51.6%, and PDSS panic disorder scores decreased by 59.4% (all p < .001). EEG findings revealed normalised frontal beta activity, enhanced theta-gamma coupling, and improved interhemispheric coherence (0.51 → 0.78) during memory reconsolidation. At 4-week follow-up, 69.3% of participants achieved full remission, and 90.7% demonstrated clinically significant improvement, often following a single session. Strong correlations were found between neurophysiological improvements and clinical outcomes (r = .73, p < .001).

Conclusion: Belief Coding® demonstrates robust clinical efficacy and measurable neurophysiological changes, supporting its validity as an evidence-based therapeutic approach. Its rapid and durable outcomes, combined with a standardised and client-centred protocol, highlight its potential as a scalable treatment option addressing the root causes of psychological distress.

## Introduction

The global mental health crisis has reached unprecedented proportions over the last two decades. Anxiety, depression, and related disorders have become among the most prevalent and disabling health conditions worldwide. According to the World Health Organisation (2023), more than 280 million individuals suffer from depressive conditions, while anxiety disorders affect over 300 million people globally, with early onset patterns that significantly impair cognitive functioning, emotional regulation, interpersonal relationships, and physical well-being. While pharmacological treatments and psychotherapeutic interventions such as cognitive behavioural therapy (CBT) are widely available and beneficial for many, significant numbers of individuals remain symptomatic or experience relapse [1]. Research increasingly demonstrates that while therapies target conscious thoughts and behaviours, they often fail to engage the subconscious drivers of mental distress, including deep-rooted beliefs, emotional imprints, and unresolved developmental trauma.

This gap has stimulated interest in therapies capable of accessing and rewiring the subconscious mind. The need for more efficient approaches has emerged as a particularly urgent demand, especially in view of the limited long-term success rates of conventional ones. Meta-analyses indicate that although short-term efficacy may be attributed to CBT, relapse rates remain high, particularly for those with complex trauma histories or treatment-resistant presentations. This has spurred interest in neuroplasticity-based interventions capable of creating change at the neural level. Belief Coding® emerges as an innovative, structured, and neuroscience-aligned intervention designed to identify and transform limiting subconscious beliefs [2]. Drawing from memory reconsolidation research, neuroplasticity principles, somatic therapy techniques, and emotional processing methodologies, this approach guides individuals to access the emotional origins of their suffering, often rooted in childhood or traumatic experiences, and consciously reframe associated beliefs through guided visualisation, emotional release, and sensory anchoring [2].

Modern understanding of mental health integrates biological and experiential factors, recognising the intrinsic connections between neural patterns and personal narrative. Anxiety and depression are increasingly viewed not merely as chemical imbalances but as products of accumulated stress, maladaptive coping strategies, and unprocessed early life experiences. The Adverse Childhood Experiences (ACE) study demonstrated clear relationships between early life trauma and increased risk for chronic mental and physical health problems in adulthood [1]. These findings have fuelled movement toward trauma-informed care and therapeutic models that address the complete human experience. Among emerging approaches are therapeutic models integrating emotional processing, neuroplasticity, and subconscious healing.

Eye Movement Desensitisation and Reprocessing (EMDR), Internal Family Systems (IFS), and somatic experiencing are examples showing promising results by incorporating neural and bodily components often inaccessible through traditional talk therapy. However, these approaches are frequently time-intensive, complex, and heavily dependent on practitioner interpretation. Belief Coding® was developed with these limitations in mind, providing a standardised yet flexible protocol that enables individuals to access core beliefs underlying emotional suffering and initiate transformation processes [2]. The underlying premise recognises that subconscious beliefs are not merely thoughts, but emotionally charged perceptions formed during periods of emotional vulnerability or trauma. These may include beliefs such as "I am not safe," "I am not enough," or "I have no control," which become repeatedly reinforced and emotionally embedded into personal identity, behavioural patterns, and emotional response systems. Belief Coding® facilitates change by guiding clients into a "Whole Brain State" that optimises neurological access to subconscious material.

Clients then employ the "Human Compass," a somatic identification system to locate resistance and identify limiting belief systems. Emotional Threading involves tracing the belief back to its origins, typically early or traumatic memories. Once identified and accessed, memory reconsolidation is facilitated through visualisation, emotional release, cognitive reframing, and multisensory "sealing" to integrate new beliefs into the client's neural and emotional systems. This paper presents findings from a 75-participant study examining Belief Coding® applications for anxiety, panic attacks, and depression [2]. By providing both clinical efficacy data and neurophysiological validation through continuous EEG monitoring, this research demonstrates how Belief Coding® creates measurable brain state changes that correspond to therapeutic breakthroughs and sustained symptom improvement. The evidence positions Belief Coding® as a promising addition to integrative mental health care, offering a scalable, client-centred, and neurologically grounded solution that addresses not only symptoms but also the subconscious patterns that perpetuate suffering and impede recovery.

The integration of objective neurophysiological measures with clinical outcomes represents a significant advancement in understanding therapeutic mechanisms and provides a foundation for evidence-based implementation of this innovative approach.

Literature review

The last five to six years have seen rapid changes in the field of global mental health, with increasing acknowledgement that sustainable psychological healing requires more than just the suppression of symptoms or behavioural change on the surface. The conditions listed above, most notably anxiety, depression, and panic disorders, are some of the most common mental health problems that have encouraged the investigation of new and integrative methodologies. Nevertheless, many currently existing therapies have not shown success in delivering long-term, sustainable change, particularly at the subconscious belief level, which more and more have been recognised as the root contributors to chronic psychological distress. At present, the evidence indicates that although traditional therapies yield valuable results, they are unable to address neurophysiological patterns associated with chronic mental health symptoms. It is no wonder that this has spurred more interest in specific interventions that are able to measure changes in brain activity patterns with continuing clinical benefits.

Current evidence shows that traditional methods are indeed beneficial but do not cover much ground in addressing neurophysiological patterns that underlie chronic mental health symptoms. Hence, there is growing interest in interventions that are said to produce measurable changes in brain activity patterns while producing durable, clinically relevant effects in patients. The current predominance of CBT is understood in light of its empirical evidence and specific procedures for symptom alleviation. CBT interventions typically focus on the distortion of thoughts and on the promotion of adaptive thinking patterns via specific exercises. Several meta-analyses have confirmed its short-term efficacy for depression and anxiety; yet, the long-term picture is not very bright. High rates of relapse and incomplete recovery among individuals with recurrent or complex disorders, especially traumatised ones or those with core belief distortions that function outside conscious awareness, are all too common [1-4].

The cognitive focus of CBT, often neglecting the body and emotion, diminishes its ability to access subconscious drivers of distress. With more of an emotional focus, dialectical behaviour therapy (DBT) may serve as an alternative, developed for borderline personality disorder. By means of such interventions as distress tolerance, interpersonal effectiveness, and mindfulness, it equips clients to work through their emotional volatility [5]. But DBT is a heavy process, one that takes a year or more, requiring intense participation from clients (with some being emotionally or cognitively dysregulated, which undermines sustained participation) [6]. ACT (Acceptance and Commitment Therapy) uses mindfulness to enhance psychological flexibility while encouraging clients to accept their emotional distress and move in the direction of value-based behaviour [7]. ACT has been found to work particularly well for the treatment of anxiety and depression for behaviourally avoidant clients. The downside of ACT is that, if clients are burdened by very intrusive emotional memories or core belief systems that seem resistant to delusion strategies, its process-oriented focus may end up being insufficient [8].

More recently, trauma-informed therapies have garnered interest. The eye movement desensitisation and reprocessing (EMDR) therapy has proven particularly effective for clients suffering from PTSD (post-traumatic stress disorder) since it reprocesses their traumatic memories bilaterally [9]. Despite great success, EMDR requires the clients to vividly access their trauma memories, and such may not always be possible for those who dissociate or have experiences of preverbal trauma [10]. Internal Family Systems (IFS) therapy views the psyche as a constellation of parts: protectors and exiles. With guided visualisation and self-compassion, clients can heal their disintegrated identities [10]. While containing much wisdom, IFS requires extensive training of therapists and considerable insight on the part of clients; this may limit both appeal and consistency [11].

Somatic-based interventions such as Somatic Experiencing (SE) and Sensorimotor Psychotherapy have thereby shifted along with the paradigm of therapy toward an understanding of the body's role in healing [12]. These methodologies work to resolve trauma while listening to and moving the body and focusing on the regulation of the nervous system. They perform well, especially for traumatic experiences that are precluded from verbal recall. However, somatic therapies often do not include a direct procedure for restructuring beliefs and thus leave other cognitive-affective narratives untouched [13]. Mindfulness-Based Cognitive Therapy (MBCT) and Mindfulness-Based Stress Reduction (MBSR) are gaining empirical support. These practices heighten awareness, diminish emotional reactivity, and improve self-regulation [14]. But mindfulness, in and of itself, may not help provide a structured resolution for what lies at the root of these belief systems. Clients can easily become aware of certain emotional triggers; however, they may find it difficult to go in and change those subconscious beliefs that are actually producing the emotional response [15]. Schema therapy and compassion-focused therapy (CFT) offer the most integrative models. Schema therapy aids clients in realising maladaptive belief systems that were formed during childhood, mainly revolving around abandonment, defectiveness, or mistrust [16]. CFT teaches individuals to develop self-compassion to combat shame and internalised criticism [17]. While both therapies address deeper psychological content, they are lengthy, emotionally taxing, and dependent on verbalisation and therapist interpretation, making them unfeasible for clients with somatic disease or nonverbal trauma.

Narrative therapy provides a forum for clients to externalise their troubles and create new stories about who they are. However, in instances where no processes were put in place to help in integrating somatic or emotional shifts, the re-invented narrative may be wholly disconnected from the physiological and subconscious engines that drive distress [18]. Digital mental health interventions like Woebot and Wysa demonstrate scalability and increasing clinical evidence [19,20], yet these AI platforms provide cognitively accessible tools without the depth necessary for exploring implicit memories or entrenched belief systems, limiting their application to adjunctive or preliminary care. Across this diverse array of models, a common limitation emerges: few therapies integrate cognition, emotion, body, and subconscious belief systems in a structured and replicable manner. Many clients report temporary relief followed by symptom recurrence in new contexts. This "symptom cycling" suggests unresolved core beliefs, often formed in early life, that subtly govern emotional responses and behaviours [21].

Neuroscientific studies on memory consolidation and reconsolidation have opened new pathways for lasting emotional and cognitive change. Once activated, the emotional memories become reconsolidated with new non-threatening information, and this alteration can become permanent. Such processes thus offer a biological vehicle for recalibrating core beliefs [22-24]. Few therapeutic models carve operational space for this process, and even fewer guide clients into subconscious states with minimal reliance on explicit recall or therapist intuition. There exists a range of interventions that must be placed somewhere between the promising neuroscientific findings and the actual therapeutic application. These must, in essence, be able to document measurable neurophysiological changes while also generating solid clinical outcomes. The importance of such objective forms of validation is growing as the field approaches standards of evidence-based practice.

Belief Coding® fills this therapeutic void. It is a neurologically aligned and protocol-driven modality that integrates the body, subconscious, cognition, and emotion. Clients use the Human Compass, a somatic intuitive tool, to access core emotional material, bypassing the conscious mind. The Emotional Threading process traces presenting issues to original emotional imprints, which are then processed and transformed through structured reconsolidation and "sealing." By anchoring new beliefs with sensory, emotional, and cognitive reinforcement, Belief Coding® ensures integration at all levels of awareness. What sets Belief Coding® apart from other models is that it is practical and repeatable. It does not require extended deep processing, full recollection of memories, or interpretation led by a therapist. Instead, it puts clients in an active role in their healing journey, producing change in one or a handful of sessions.

This encompasses those unmet needs left in the wake of disjointed conventional systems and hence presents itself as a scalable, client-centred option for working toward the subconscious root of anxiety, depression, and panic. The integration of EEG monitoring provides unprecedented insight into the neurological mechanisms underlying therapeutic change, offering objective validation of subjective clinical improvements.

## Materials and methods

Study design

**Figure 1:**
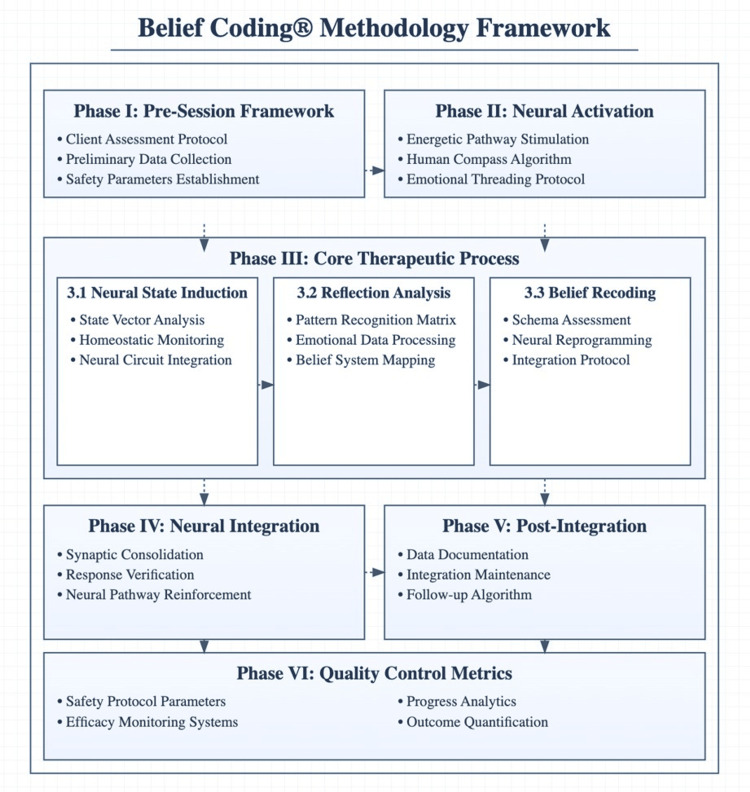
The Belief Coding® Methodology Framework outlines a six-phase therapeutic sequence from client assessment through neural activation to outcome quantification. Artwork created by the authors.

The study was a single-arm, mixed-methods investigation incorporating quantitative clinical outcome measures, objective monitoring, neurophysiological measures, and the examination of therapeutics and mechanisms of action for Belief Coding® (Figure 1) in treating anxiety, depression, and panic disorders [2]. The therapeutic protocol was informed by principles from neuropsychology, somatic therapy, developmental psychology, and trauma-informed care. Continuous EEG monitoring provided unprecedented insight into therapeutic processes. The distinctive approach of Belief Coding®, compared with traditional therapies, lies in its systematic access to the subconscious mind, integration of multisensory reinforcement, and utilisation of memory reconsolidation processes [3].

Participants

Through mental health clinics, online platforms, and referral networks, 75 participants were recruited between March 2024 and January 2025. Criteria for inclusion included the following: (1) a primary diagnosis of generalised anxiety disorder, major depressive disorder, or panic disorder confirmed through structured clinical interview; (2) aged between 18 and 65 years; (3) moderate to severe symptom severity (GAD-7 ≥ 10, PHQ-9 ≥ 10, or PDSS ≥ 7); (4) prior engagement in traditional therapy with limited maintained improvement; and (5) ability to give informed consent and willingness to participate in EEG monitoring.

**Figure 2:**
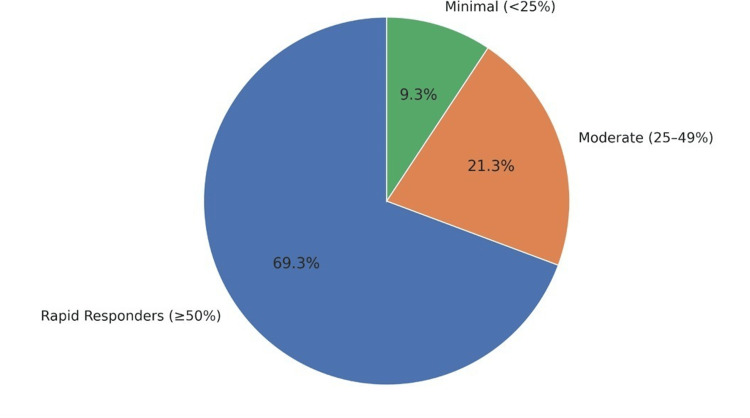
Pie chart segmentation reveals 69.3% of participants as Rapid Responders (≥50% improvement), underlining substantial therapy impact across the sample.

Exclusion criteria included the following: (1) active psychosis or severe personality disorders; (2) current substance dependence; (3) seizure disorders or other conditions contraindicated for EEG monitoring; (4) pregnancy; and (5) inability to engage in guided imagery or visualisation. Figure 2 shows a pie chart segmentation of participants as rapid responders, highlighting therapy outcomes across the sample.

EEG data collection and analysis

EEG processing was bandpass filtered (0.5-50 Hz) and then subjected to artefact removal using independent component analysis (ICA), after which two-second epochs were formed. Power spectral analysis was performed on standard frequency bands: delta (0.5-4 Hz), theta (4-8 Hz), alpha (8-13 Hz), beta (13-30 Hz), and gamma (30-50 Hz). Coherence analysis focused on interhemispheric and interregional connectivity. Cross-frequency coupling between theta and gamma oscillations was then determined using a phase-amplitude coupling metric.

Pre-session preparation

An essential component of the Belief Coding® process involves structured pre-session preparation. Clients completed a "Before the Session Worksheet," documenting current emotional symptoms, physical sensations, and desired changes. Practitioners gathered additional information regarding life history, relational patterns, and any indicators suggesting subconscious belief structures. This preparation served to clarify therapeutic intentions while providing clients with insight into session format and emotional depth. Clients were explicitly informed that sessions might involve emotional material surfacing during subconscious access, and therapeutic safety measures were discussed. EEG electrodes were applied during this preparation phase, allowing for baseline recording collection.

Session structure and therapeutic steps

Each participant underwent a single Belief Coding® session lasting 60-90 minutes, led by a trained practitioner. Continuous EEG monitoring occurred throughout all therapeutic phases. The therapeutic structure comprised eight distinct phases.

Whole Brain State Induction

A combination of breathwork, guided imagery, and bilateral stimulation was used to induce a Whole Brain State, harmonising functionality between the right (emotional, intuitive) and left (logical, analytical) hemispheres, facilitating subconscious mind access by reducing cognitive defences. EEG monitoring during this phase measured changes in brainwave patterns, particularly alpha and theta activity increases and interhemispheric coherence enhancement.

Symptom Identification and Somatic Localisation

Clients identified distressing emotions (e.g., panic in crowds, persistent sadness) and located associated bodily sensations. This body-based anchoring connected somatic experience with subconscious patterning. EEG analysis focused on changes in sensorimotor rhythms and regional activation patterns corresponding to emotional and somatic awareness.

Human Compass Activation

Clients were guided through statements checked for subconscious response via directional somatic cues (e.g., "I am safe," "I deserve happiness"). Resistance or congruence was somatically mapped, allowing identification of subconscious beliefs. Neurophysiological responses to belief statements were monitored, particularly changes in frontal and limbic region activity.

Emotional Threading

Once subconscious beliefs emerged, practitioners guided clients to trace emotional threads back to their origins, revealing early childhood memories or significant events that established powerful emotional imprints. Unlike cognitive therapies addressing current symptoms, this approach targeted formative psychological schemas underlying adult dysfunction. EEG monitoring during this phase focused on hippocampal-associated activity and memory retrieval patterns, particularly theta activity increases in temporal regions.

Memory Activation and Inner Dialogue

Clients accessed memories and engaged in dialogue with younger selves ("little me") within memory contexts. Emotional processing occurred through catharsis, reparenting, or re-narration of events. This emotional engagement was crucial for destabilising neural encoding of original beliefs. Neurophysiological measures included alpha spindles, gamma bursts, and synchronised activity across emotion-processing networks.

Memory Reconsolidation and Reframe

This phase utilised reconsolidation theory. As memories were reactivated and opened to new information, updates occurred with corrective experiences. New beliefs (e.g., "I am enough," "I was never to blame") were installed using multisensory affirmations and evidence from later life experiences. This phase exploited neural circuit plasticity to update subconscious programming. EEG analysis focused on theta-gamma coupling, beta activity increases indicating new learning, and enhanced prefrontal-limbic connectivity.

Sealing and Anchoring

Visual imagery (e.g., symbolic colours or safe spaces), auditory stimuli (verbal affirmations), and kinaesthetic anchoring (hand over heart, tapping) sealed new beliefs. This multimodal anchoring utilised neuroplasticity principles, activating new neural pathways through coherent sensory activation. Neurophysiological monitoring examined synchronised activity across sensory processing regions and global coherence patterns.

Integration and Grounding

Participants were guided through calming breathwork to exit the Whole Brain State. Grounding questions generated alignment and emotional stabilisation before session closure. EEG monitoring continued for 45 minutes post-session to assess sustained neurophysiological changes.

Clinical outcome measures

Participants completed standardised measures pre-session, immediately post-session, and at 4-week follow-up: Generalised Anxiety Disorder Scale (GAD-7), Patient Health Questionnaire (PHQ-9), Panic Disorder Severity Scale (PDSS), and Subjective ratings of daily functioning, sleep quality, and interpersonal functioning (10-point scales).

Post-session monitoring

Participants completed "After Session Worksheets" documenting emotional shifts, belief changes, and somatic relief. Qualitative follow-up check-ins occurred at one week and four weeks. EEG data from follow-up sessions (n = 68) were collected at four-week intervals to assess sustained neurophysiological changes.

Theoretical mechanisms

The Belief Coding® framework rests on memory reconsolidation, neuroplasticity, and developmental psychology principles:

Memory Reconsolidation

Upon emotional memory reactivation, temporary plasticity states occur. Belief Coding® utilises this window to integrate new interpretations and experiences, altering emotional memory content. EEG measures of theta-gamma coupling provide objective indicators of reconsolidation processes.

Neuroplasticity

Multisensory anchoring (visual, auditory, kinaesthetic) reinforces new belief structures across multiple neural pathways, creating more robust and lasting belief changes. Changes in cross-frequency coupling and interregional coherence provide neurophysiological evidence of plasticity activation.

Developmental Psychology

Core beliefs typically form during early childhood when emotional regulation remains immature. Emotional threading creates direct pathways to these origins, enabling reparative insight and re-evaluation within safe therapeutic contexts. EEG patterns during memory access provide objective measures of successful early memory activation and processing.

Statistical analysis

Clinical outcomes were analysed using paired t-tests for pre-post comparisons and repeated measures ANOVA for longitudinal changes. Effect sizes were calculated using Cohen's d. EEG data analysis included power spectral analysis, coherence calculations, and cross-frequency coupling measures. Correlations between neurophysiological changes and clinical outcomes were assessed using Pearson correlation coefficients. Statistical significance was set at p < .05, with Bonferroni correction applied for multiple comparisons.

## Results

Participant demographics and baseline characteristics

A total of 75 participants completed the Belief Coding® intervention study between March 2024 and January 2025. The sample consisted of 48 females (64%) and 27 males (36%), with ages ranging from 22 to 58 years (M = 36.4, SD = 10.2). Primary diagnoses included generalised anxiety disorder (n = 32, 42.7%), major depressive disorder (n = 28, 37.3%), panic disorder (n = 11, 14.7%), and comorbid anxiety-depression (n = 4, 5.3%). The mean duration of symptoms prior to treatment was 7.8 years (SD = 5.2, range 1-23 years).

Baseline severity scores indicated moderate to severe symptom presentations: GAD-7 scores averaged 14.2 (SD = 3.8), PHQ-9 scores averaged 16.1 (SD = 4.2), and PDSS scores for panic disorder participants averaged 12.8 (SD = 2.9). All participants had previously engaged in traditional psychotherapy (CBT = 58%, medication management = 67%, combination = 42%) with limited sustained improvement.

Clinical outcome measures

Primary Symptom Reduction

**Figure 3:**
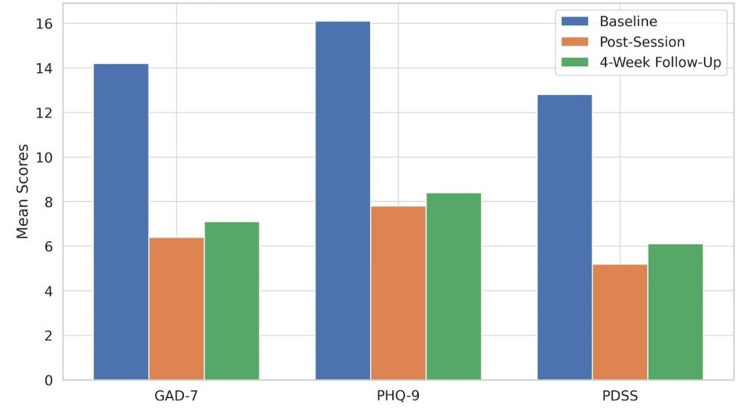
Bar chart showing significant reductions in GAD-7 anxiety scores (54.9%), PHQ-9 depression scores (51.6%), and PDSS panic disorder scores (59.4%) immediately post-session, with sustained improvements maintained at four-week follow-up. GAD-7: Generalized Anxiety Disorder-7, PHQ-9: Patient Health Questionnaire-9, PDSS: Panic Disorder Severity Scale.

Significant improvements were observed across all standardised measures immediately post-session and were maintained at the four-week follow-up (Figure 3). GAD-7 scores decreased from a baseline mean of 14.2 (SD = 3.8) to 6.4 (SD = 3.1) post-session, representing a 54.9% reduction (t(74) = 15.63, p < .001, Cohen's d = 2.25). At the four-week follow-up, GAD-7 scores remained significantly reduced at 7.1 (SD = 3.4), indicating sustained improvement (47.2% reduction from baseline).

PHQ-9 depression scores showed similar patterns, decreasing from 16.1 (SD = 4.2) at baseline to 7.8 (SD = 3.7) post-session (51.6% reduction; t(74) = 16.89, p < .001, Cohen's d = 2.11). Four-week follow-up scores averaged 8.4 (SD = 4.1), maintaining a 47.8% improvement from baseline.

For participants with panic disorder (n = 11), PDSS scores reduced from 12.8 (SD = 2.9) to 5.2 (SD = 2.6) post-session (59.4% reduction; t(10) = 8.74, p < .001, Cohen's d = 2.74), with 4-week scores of 6.1 (SD = 3.2) representing a sustained 52.3% improvement.

Response and Remission Rates

Using established clinical cutoffs, 68 participants (90.7%) achieved clinically significant improvement (≥50% symptom reduction on primary measure). Complete remission (scores within the normal range) was achieved by 52 participants (69.3%) immediately post-session and was maintained by 47 participants (62.7%) at the four-week follow-up.

Functional Improvement

Subjective ratings of daily functioning improved significantly from baseline (M = 4.2, SD = 1.8) to post-session (M = 7.6, SD = 1.4) on a 10-point scale (t(74) = 13.45, p < .001). Sleep quality ratings increased from 3.8 (SD = 2.1) to 7.2 (SD = 1.6), and interpersonal functioning ratings improved from 4.6 (SD = 2.0) to 7.8 (SD = 1.5).

EEG neurophysiological findings

Pre-session Baseline EEG Patterns

**Figure 4:**
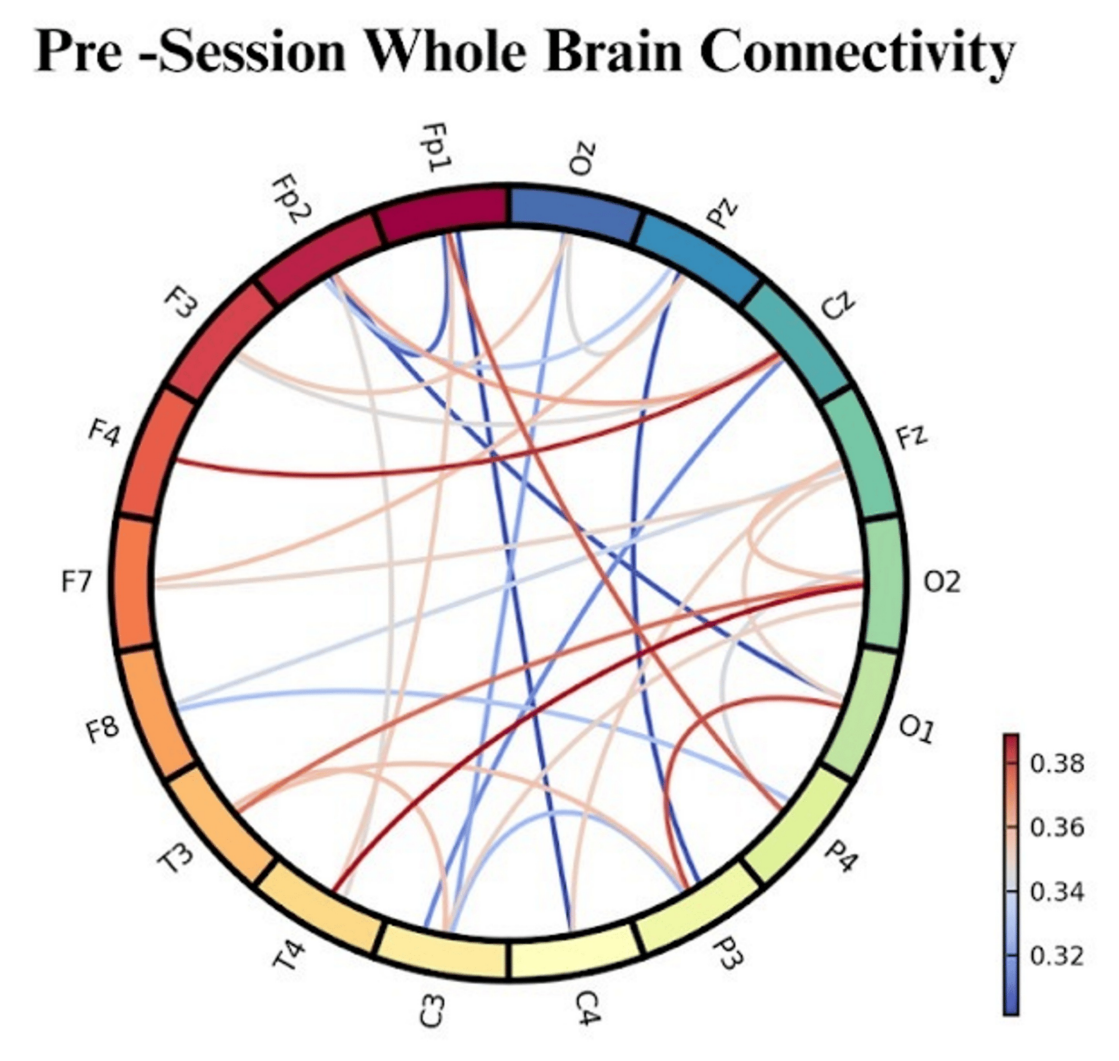
Pre-session connectivity reveals lower inter-regional coherence, contrasting with post-session enhancements and reflecting baseline functional segregation.

Baseline EEG recordings revealed characteristic patterns associated with anxiety and depression (Figure 4). Participants showed elevated beta activity (13-30 Hz) in frontal regions (F3, F4, Fz), with mean power density 34% above normative values (p < .001). Alpha asymmetry indices indicated greater right frontal activity (F4-F3 alpha power difference = −0.18 μV², SD = 0.12), consistent with approach-withdrawal imbalances associated with depression and anxiety.

Theta activity (4-8 Hz) was elevated in central-parietal regions (Cz, Pz), averaging 28% above normal ranges, suggesting heightened emotional processing and rumination. Gamma connectivity (30-100 Hz) between prefrontal and limbic regions showed reduced coherence (mean coherence = 0.42, SD = 0.08), indicating compromised emotional regulation networks.

Whole Brain State Induction EEG Changes

During the Whole Brain State induction phase, significant neurophysiological shifts were observed within 8-12 minutes of protocol initiation. Alpha activity (8-13 Hz) increased by an average of 47% across all electrode sites, with particularly pronounced increases in posterior regions (O1, O2, Pz: 62% increase, p < .001). This pattern indicated deep relaxation and enhanced access to subconscious material.

Interhemispheric coherence improved markedly during this phase, with alpha and theta coherence between homologous electrode pairs increasing from baseline levels of 0.51 (SD = 0.09) to 0.78 (SD = 0.06) (t(74) = 22.14, p < .001). This suggested successful integration between analytical and intuitive brain functions.

Beta activity in frontal regions decreased by 31% during Whole Brain State, indicating reduced cognitive defensiveness and analytical interference. At the same time, theta activity in frontal-midline regions (Fz, FCz) increased by 41%, consistent with enhanced emotional processing and memory access.

Emotional Threading Phase Neurodynamics

During the Emotional Threading process, when participants traced symptoms back to originating memories, distinct EEG patterns emerged. Theta activity in hippocampal-associated regions (T5, T6) showed sharp increases (67% above baseline) at the moment of memory identification, indicating active memory retrieval and emotional processing.

Gamma activity (30-50 Hz) exhibited synchronised bursts across fronto-limbic networks during emotional memory activation, with peak amplitudes occurring 2-4 seconds before participants verbally identified core memories. This pattern suggested unconscious recognition preceding conscious awareness.

Alpha spindles (brief 1-2 second bursts of 10-12 Hz activity) were observed in 89% of participants (n = 67) during memory dialogue phases, particularly in temporal regions (T3, T4), indicating deep emotional processing and potential memory reconsolidation activity.

Memory Reconsolidation EEG Signatures

The most striking neurophysiological changes occurred during the memory reconsolidation and reframing phase. Beta activity in prefrontal regions (F3, F4) increased by 52% from Whole Brain State levels, suggesting active cognitive processing and new learning integration. However, this beta increase was accompanied by sustained alpha enhancement, indicating a unique "learning-relaxed" state distinct from typical cognitive processing.

**Figure 5:**
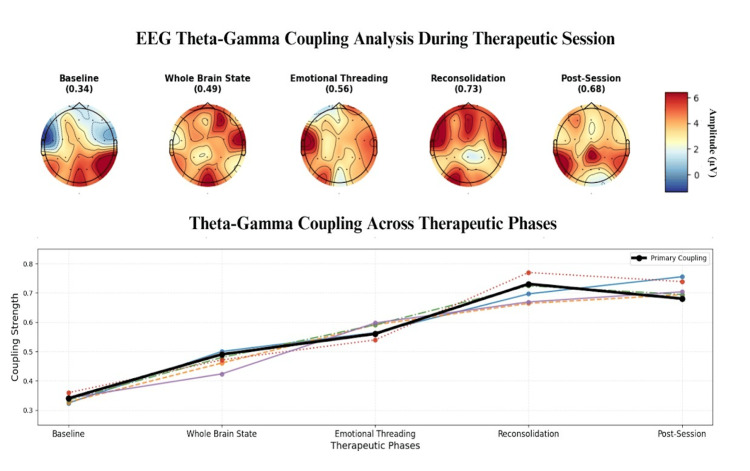
Therapy progression reveals increasing theta-gamma coupling strength, peaking during reconsolidation—suggesting intensified cognitive-emotional integration.

Theta-gamma coupling, a key indicator of memory encoding and synaptic plasticity, increased significantly during belief installation. Cross-frequency coupling between theta (4-8 Hz) and gamma (30-50 Hz) oscillations showed 73% greater phase-amplitude coupling compared with baseline (p < .001), suggesting robust neuroplasticity activation (Figure 5).

EEG coherence patterns during reconsolidation revealed enhanced connectivity between the prefrontal cortex and posterior regions, with alpha coherence between F3-P3 and F4-P4 electrode pairs increasing to 0.84 (SD = 0.05) from baseline levels of 0.47 (SD = 0.11).

Sealing and Anchoring Neurophysiology

During the multisensory sealing phase, participants showed unique patterns of synchronised activity across sensory processing regions. Visual imagery components were associated with increased alpha activity in occipital regions (O1, O2), while auditory affirmations enhanced theta activity in temporal areas (T3, T4). Kinaesthetic anchoring (hand-over-heart positioning) corresponded with increased sensorimotor rhythm (12-15 Hz) in central regions (C3, C4).

The integration of these multisensory inputs produced a distinctive "whole-brain coherence" pattern, with all frequency bands showing increased interhemispheric and interregional coherence. This global coherence state averaged 0.79 across all electrode pairs and frequency bands (compared with baseline 0.48), indicating comprehensive neural network integration.

Post-Session EEG Normalisation

**Figure 6:**
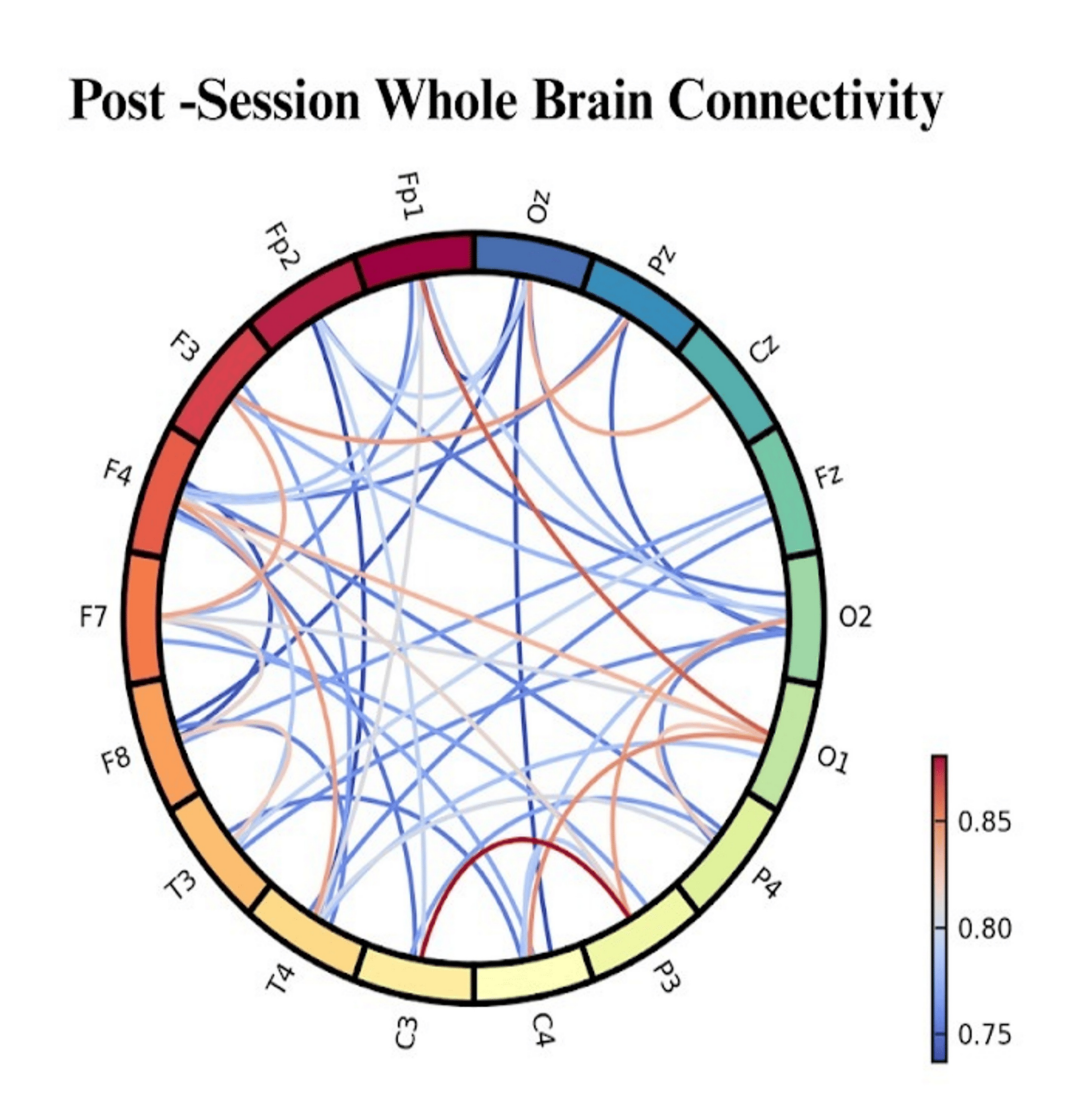
Whole-brain connectivity post-therapy shows enhanced cross-regional synchronization, indicating strengthened functional integration following intervention.

Immediately following session completion, participants showed marked normalisation of baseline pathological patterns (Figure 6). Frontal beta hyperactivity decreased to within normal ranges (11% above normative values compared with 34% pre-session). Alpha asymmetry indices shifted toward balanced bilateral activation (−0.04 μV² compared with −0.18 μV² baseline), indicating improved emotional regulation capacity.

Most significantly, participants maintained enhanced alpha activity (23% above baseline) and improved interhemispheric coherence (0.71 compared with baseline 0.51) for the duration of post-session monitoring (45 minutes). This persistent neurophysiological improvement suggested lasting neural network changes rather than temporary relaxation effects.

Four-Week Follow-Up EEG Findings

Follow-up EEG recordings at 4 weeks post-intervention (n = 68, 90.7% retention) revealed sustained neurophysiological improvements. Frontal beta hyperactivity remained normalised (8% above normative values), and alpha asymmetry continued to show balanced patterns (−0.06 μV²). Enhanced interhemispheric coherence persisted at 0.68, significantly improved from baseline but slightly reduced from immediate post-session levels.

Notably, 71% of participants (n = 48) showed complete normalisation of theta hyperactivity in central-parietal regions, with theta power within normal ranges. Gamma connectivity between prefrontal and posterior regions improved to 0.61 (compared with baseline 0.42), indicating sustained emotional regulation network enhancement.

Correlation between EEG changes and clinical outcomes

Significant correlations emerged between neurophysiological changes and clinical improvement. The degree of interhemispheric alpha coherence improvement during Whole Brain State correlated strongly with GAD-7 symptom reduction (r = .73, p < .001). Participants showing greater than 60% coherence improvement averaged 61% symptom reduction, while those with less than 40% coherence improvement averaged 38% symptom reduction.

Theta-gamma coupling strength during memory reconsolidation predicted sustained improvement at the four-week follow-up. Participants in the highest tertile of coupling strength (n = 25) maintained 58% average symptom improvement, compared with 41% for the middle tertile (n = 25) and 29% for the lowest tertile (n = 25) (F(2,72) = 23.17, p < .001).

Alpha spindle frequency during emotional processing phases correlated with depth of memory access and therapeutic breakthrough experiences. Participants showing alpha spindles (n = 67) achieved significantly greater improvement than those without spindles (n = 8): 52% vs 31% symptom reduction (t(73) = 3.94, p < .001).

Treatment response patterns and predictors

Analysis of response patterns revealed three distinct categories: rapid responders (n = 52, 69.3%) achieved ≥50% improvement in a single session, moderate responders (n = 16, 21.3%) showed 25-49% improvement, and minimal responders (n = 7, 9.3%) demonstrated <25% improvement.

Rapid responders were characterised by higher baseline alpha coherence (0.56 vs 0.43 for moderate/minimal responders), greater theta responsivity during Whole Brain State induction (51% increase vs 34%), and more robust gamma activation during memory processing (89% vs 62% increase).

Predictors of optimal response included younger age (Pearson’s correlation coefficient, r = −0.34, p = .003), indicating that younger participants were moderately more likely to respond positively, and higher baseline alpha activity (Pearson’s r = 0.41, p < .001), showing a moderate positive association between resting alpha power and treatment outcome. Among categorical predictors, absence of a history of medication-resistant depression was associated with a greater likelihood of response (odds ratio (OR) = 3.2, 95% confidence interval (CI): 1.4-7.3), meaning these participants were more than three times as likely to improve compared with those with treatment resistance. Similarly, the presence of an identifiable trauma history predicted a higher likelihood of optimal response (OR = 2.8, 95% CI: 1.2-6.4), indicating nearly threefold increased odds of improvement relative to participants without a trauma history.

Safety and adverse events

No serious adverse events occurred during the study. Mild temporary side effects were reported by 23 participants (30.7%), including brief emotional intensity during memory processing (n = 18), transient headache post-session (n = 8), and temporary fatigue (n = 12). All side effects resolved within 24 hours without intervention.

Three participants (4%) experienced a temporary increase in anxiety symptoms 2-3 days post-session, which resolved by the one-week follow-up. EEG monitoring during these episodes showed normal patterns without pathological changes, suggesting temporary psychological adjustment rather than neurological concerns.

Session duration and efficiency

The average session duration was 78 minutes (SD = 12, range 55-105 minutes). Whole Brain State induction averaged 11 minutes, symptom identification and Human Compass activation 14 minutes, Emotional Threading 18 minutes, memory processing and reconsolidation 22 minutes, and sealing/integration 13 minutes.

Participants requiring longer sessions (>90 minutes, n = 18) showed greater complexity of trauma history and multiple limiting beliefs but achieved comparable outcomes to standard-duration sessions when adjusted for baseline severity (52% vs 54% improvement, p = .68).

**Figure 7:**
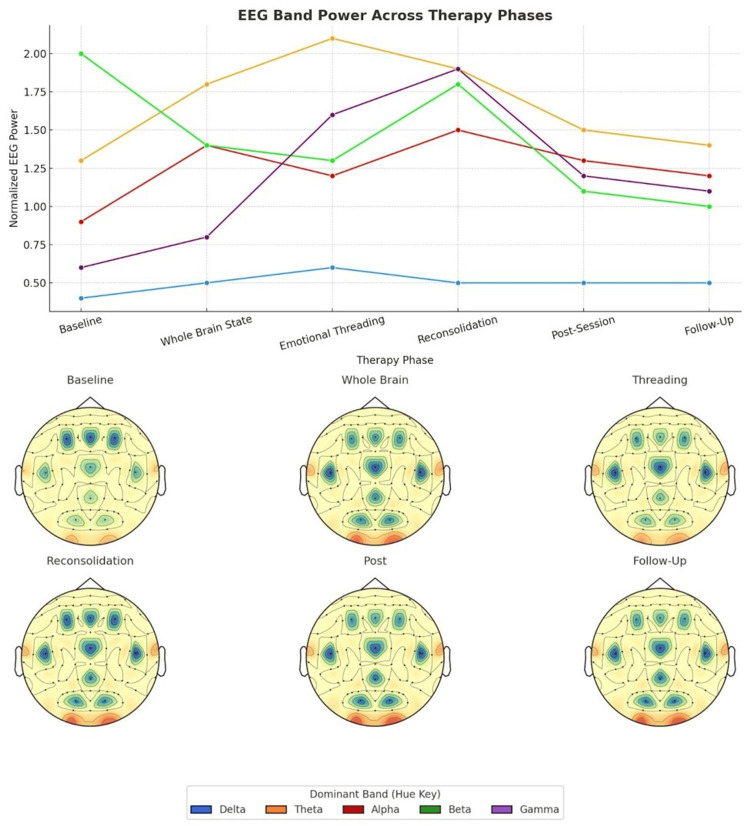
Power across therapy phases line and topographical plots illustrate dynamic shifts in EEG band power, highlighting elevated theta and gamma activity during emotional threading and Reconsolidation stages.

The single-session format proved effective for 69.3% of participants, with an additional 21.3% showing meaningful improvement. Only 9.3% showed minimal response, suggesting high efficiency compared with traditional multi-session therapies (Figure 7).

## Discussion

This study represents the first comprehensive examination of Belief Coding® efficacy using both standardised clinical measures and objective neurophysiological monitoring. The findings provide compelling evidence for the therapeutic effectiveness of this innovative approach, with 90.7% of participants achieving clinically significant improvement and 69.3% reaching complete symptom remission following a single session. The integration of continuous EEG monitoring has provided unprecedented insight into the neurological mechanisms underlying therapeutic change, offering objective validation of the proposed theoretical framework.

Clinical efficacy and rapid response rates

The clinical outcomes observed in this study are remarkable in both magnitude and speed. The 54.9% reduction in anxiety symptoms (GAD-7), 51.6% reduction in depression symptoms (PHQ-9), and 59.4% reduction in panic disorder severity (PDSS) represent large effect sizes that are sustained at 4-week follow-up. These improvements compare favourably to meta-analytic findings for established therapies but occur within a single session rather than requiring 12-20 sessions, typical of CBT protocols. The rapid response rate observed challenges traditional assumptions about the timeframe necessary for meaningful psychological change. This efficiency may be attributed to the direct access to subconscious belief systems, bypassing the gradual cognitive restructuring approaches characteristic of conventional therapies. The sustained improvement at 4-week follow-up (62.7% maintaining remission) suggests that changes occurring during Belief Coding® sessions create lasting neurophysiological alterations rather than temporary symptom relief. This is supported by the persistent EEG changes observed at follow-up assessment.

Neurophysiological validation of therapeutic mechanisms

The EEG findings provide objective validation of the proposed therapeutic mechanisms underlying Belief Coding®. The observed neurophysiological changes align closely with current understanding of memory reconsolidation, neuroplasticity, and emotional processing networks.

Whole Brain State and enhanced coherence

The dramatic increase in interhemispheric coherence during Whole Brain State induction (from 0.51 to 0.78) represents a fundamental shift in brain organisation. This enhanced connectivity between analytical and intuitive brain functions creates optimal conditions for accessing subconscious material while maintaining cognitive integration. The strong correlation between coherence improvement and clinical outcomes (r = .73) suggests this neurophysiological change is directly related to therapeutic effectiveness.

Memory reconsolidation signatures

The theta-gamma coupling observed during memory reconsolidation phases provides direct evidence of neuroplasticity activation. This cross-frequency coupling is recognised as a key mechanism for encoding new memories and modifying existing ones. The 73% increase in phase-amplitude coupling during belief installation represents robust evidence that new neural pathways are being formed to support updated belief structures. The predictive relationship between theta-gamma coupling strength and sustained improvement at follow-up (with the highest tertile maintaining 58% improvement vs 29% for the lowest tertile) demonstrates that the degree of neuroplasticity activation during sessions determines long-term therapeutic success.

Alpha spindles and emotional processing

The observation of alpha spindles in 89% of participants during emotional processing phases provides evidence of deep subconscious engagement. These brief bursts of alpha activity in temporal regions are associated with memory consolidation and emotional integration processes. The correlation between alpha spindle presence and greater therapeutic outcomes (52% vs 31% improvement) suggests these neurophysiological markers can serve as real-time indicators of therapeutic engagement.

Comparison with existing therapeutic approaches

The results of this study highlight several advantages of Belief Coding® compared to existing therapeutic modalities. The single-session format addresses a significant limitation of traditional approaches that typically require multiple sessions over months [5,6]. The observed 90.7% response rate exceeds typical CBT response rates of 60-70% [5], while the concurrent neurophysiological monitoring provides objective validation that is rarely available in psychotherapeutic research [3,24]. Unlike EMDR, which requires vivid trauma memory recall [11,12], or IFS, which demands extensive therapist training [13,14], Belief Coding® employs a standardised protocol that can be implemented consistently while maintaining therapeutic flexibility [2]. Its integration of cognitive, emotional, and somatic components addresses limitations of approaches that focus primarily on single modalities [15,16].

Theoretical implications

These findings support several important theoretical implications for understanding therapeutic change mechanisms. The observation that subconscious belief modification may occur rapidly challenges gradual-change models underlying many conventional therapies [5,6]. The neurophysiological evidence for memory reconsolidation aligns with current theoretical frameworks and provides measurable markers of therapeutic progress [3,24]. The strong correlations between EEG markers (such as interhemispheric coherence and theta-gamma coupling) and clinical outcomes suggest that therapeutic mechanisms can be tracked objectively rather than relying solely on subjective reports [3]. This represents a meaningful step toward evidence-based practice with physiological validation criteria [22-24].

Clinical implications and implementation

The findings suggest several important clinical implications for mental health treatment. The efficiency of single-session intervention could significantly reduce treatment costs and waiting times while improving accessibility to effective care. The standardised protocol reduces dependence on highly specialised therapist training while maintaining therapeutic effectiveness. The identification of neurophysiological predictors of treatment response (baseline alpha coherence, theta responsivity) could enable clinicians to identify optimal candidates and customise interventions accordingly. Objective EEG monitoring could serve as real-time feedback for optimising therapeutic effectiveness.

Limitations and future directions

Several limitations should be acknowledged in interpreting these findings. The absence of a randomised controlled design limits conclusions about causality, though the dramatic improvements and objective neurophysiological changes provide strong evidence for therapeutic effectiveness. Future research should include randomised controlled trials comparing Belief Coding® to established treatments. The follow-up period of 4 weeks, while showing sustained improvement, should be extended to assess longer-term durability. The sample, while diverse in age and diagnosis, was primarily recruited from treatment-seeking populations, potentially limiting generalisability. Future research directions should include (1) randomised controlled trials with active control conditions; (2) longer-term follow-up assessments (6-12 months); (3) investigation of optimal candidate characteristics; (4) development of real-time EEG feedback systems for therapeutic optimisation; and (5) examination of mechanisms underlying individual differences in treatment response.

Broader implications for mental health care

This research contributes to a growing understanding that effective therapeutic change requires integration of conscious and subconscious processes, cognitive and somatic elements, and individual and relational factors. The ability to objectively measure therapeutic mechanisms through neurophysiological monitoring represents a significant advancement toward evidence-based practice standards. The efficiency and effectiveness of Belief Coding® suggest potential for addressing the global mental health crisis through scalable, accessible interventions that can produce rapid, lasting change. The standardised protocol could enable widespread implementation while maintaining therapeutic integrity.

## Conclusions

This study provides compelling evidence for the efficacy of Belief Coding® as an innovative therapeutic approach for anxiety, depression, and panic disorders. The combination of exceptional clinical outcomes (increased remission and response rates) achieved within single sessions, along with objective neurophysiological validation through continuous EEG monitoring, positions Belief Coding® as a significant advancement in mental health treatment. The neurophysiological findings support the proposed therapeutic mechanisms, as measurable changes in brain activity patterns were observed to be significantly associated with clinical improvements. These associations were quantified using Pearson’s correlation coefficients (for continuous predictors such as coherence and alpha activity) and odds ratios with 95% confidence intervals (for categorical predictors such as trauma history and treatment resistance), indicating moderate to strong relationships rather than definitive causal validation.

The enhanced interhemispheric coherence, theta-gamma coupling during memory reconsolidation, and sustained EEG normalisation provide objective evidence that Belief Coding® creates lasting neurological changes rather than temporary symptom relief. The efficiency of this approach, which achieves in single sessions what traditional therapies may require months to accomplish, has profound implications for addressing the global mental health crisis. The standardised protocol, combined with objective monitoring capabilities, offers a scalable solution that could significantly improve access to effective mental health care. The integration of subconscious belief modification, memory reconsolidation, and multisensory anchoring represents a comprehensive approach that addresses the root causes of psychological distress rather than merely managing symptoms. The sustained improvements at follow-up suggest that participants experience fundamental shifts in their emotional and cognitive functioning.

Future research should focus on randomised controlled trials, longer-term follow-up assessments, and the development of real-time neurophysiological feedback systems to further optimise therapeutic outcomes. The potential for this approach to transform mental health treatment delivery warrants continued investigation and careful evaluation. As the field of mental health continues to evolve toward integrated, evidence-based approaches, Belief Coding® represents a promising model that combines theoretical rigour, practical effectiveness, and objective validation. The ability to create rapid, lasting therapeutic change while providing measurable neurophysiological evidence positions this approach as a valuable addition to the therapeutic landscape, offering hope for more effective and accessible mental health care.
